# An X-Linked Sex Ratio Distorter in *Drosophila simulans* That Kills or Incapacitates Both Noncarrier Sperm and Sons

**DOI:** 10.1534/g3.114.013292

**Published:** 2014-07-31

**Authors:** William R. Rice

**Affiliations:** * Department of Ecology, Evolution & Marine Biology, University of California, Santa Barbara, California 93106

**Keywords:** genomic conflict, SA-zygotic drive, segregation distortion, male meiotic drive, genetics of sex

## Abstract

Genomic conflict occurs when a genomic component gains a reproductive advantage at the expense of the organism as a whole. X-linked segregation distorters kill or incapacitate Y-bearing sperm, thereby gaining a transmission advantage but also reducing male fertility and generating a female-biased sex ratio. When some damaged, Y-bearing sperm survive and fertilize eggs, then the segregation distortion phenotype could be expanded by harming or killing sons in the next generation. X-linked son-killers are predicted by theory to be favored by natural selection and evolve when brothers and sisters compete for shared limiting resources and/or when brothers reduce the inclusive fitness of their sisters via sib-mating—a phenomenon called SA-zygotic drive. Here I develop and use a process-of-elimination screen to show that an unclassified X-linked sex ratio distorter (*skew*) in *Drosophila simulans* kills or incapacitates noncarrier sperm and also kills a substantial proportion of sons, *i.e.*, it has both a segregation distortion and a SA-zygotic drive phenotype. There are three unique X-linked segregation distorters known to occur in *D. simulans* named Winters, Durham, and Paris. Autosomal-dominant suppressors of Winters (*Nmy)* and Durham (*Tmy*) failed to suppress *skew*. A Y-linked suppressor of Paris, however, did suppress s*kew*, and a recombination test failed to detect recombinants between these two sex ratio distorters, indicating that they are tightly linked and plausibly identical or allelic. Son-killing may be an important yet unrecognized component of other X-linked segregation distorters.

Sex ratio distortion due to intragenomic conflict has many documented etiologies. Segregation distortion (hereafter SD, a.k.a. male meiotic drive) is a well-established form of genomic conflict that leads to biased sex ratios when the driver complex that causes SD is X- or Y-linked (reviewed in Jaenike 2001; Helleu *et al.* 2014). SD occurs in males that are heterozygotes for the driving chromosomal region when it codes for a phenotype (usually subcellular) that kills, incapacitates, or precludes the production of noncarrier sperm. SD is predicted to be especially prevalent on the sex chromosomes because the constraints for its evolution are reduced when located in regions that do not recombine between the X and Y or W and Z chromosomes (Hamilton 1967; Frank 1991; Hurst and Pomiankowski 1991). Although less commonly documented, X and Y sex chromosomes can also achieve a drive-like phenotype by feminizing genetic males (X-FEM or Y-FEM) when they have a transmission advantage that only functions during oogenesis (reviewed in chapter 3 of Burt and Trivers 2006).

Maternally transmitted cytoplasmic endosymbionts, such as *Wolbachia* and *Spiroplasma*, can contribute to another well-established form of intragenomic conflict by killing sons (hereafter son-killing or SK), *i.e.*, killing the sex that does not propagate them. This phenomenon leads to female-biased sex ratios and has been extensively studied both theoretically and empirically (reviewed in Hurst and Frost 2000; Hurst and Jiggins 2014). Cytoplasmic endosymbionts are selected to kill sons when they compete with their sisters (that do propagate the endosymbionts) for limiting resources shared among siblings and/or when sons reduce their sisters’ inclusive fitness by sib-matings that induce sufficiently strong inbreeding depression (reviewed in chapter 3 of Burt and Trivers 2006). Although less commonly documented, cytoplasmic endosymbionts have also evolved to i) sex-reverse genetically male offspring (hereafter CYTO-FEM) or ii) induce asexual reproduction in their female carriers (hereafter CYTO-ASEX) (reviewed in chapter 5 of Burt and Trivers 2006). Both of these phenotypes circumvent the dead-end transmission of the endosymbionts to males.

Paternal X chromosomes also can be selected to kill, harm, or sterilize sons (noncarriers) by the same reasoning that selectively favors SK by maternally transmitted endosymbionts. This form of intragenomic conflict—and the corresponding daughter-killing/harming process coded by the Y chromosome—has been termed sexually antagonistic zygotic drive (hereafter SA-ZD) by Rice *et al.* (2008, 2009, 2010) and is reviewed in Friberg and Rice (2014). Although SK by X-linked zygotic drivers is predicted by this theory, only one study to date provides preliminary—but not definitive— empirical evidence for this phenotype in nature (Friberg *et al.* 2011). Zygotic drive coded by the autosomes (killing noncarrier offspring irrespective of sex), however, is well documented in organisms as diverse as nematodes, mice, and beetles—and multiple nonallelic zygotic drivers have been discovered in some groups (reviewed in chapter 2 of Burt and Trivers 2006). Most of the autosomal zygotic drivers that have been identified kill noncarrier offspring via maternal-effects. However, autosomal zygotic drive via a paternal effect also has been described that kills noncarrier offspring via protein packaged in the sperm and transmitted to the zygote (Seidel *et al.* 2008, 2011). Other studies—outside the context of zygotic drive—have demonstrated that paternal effects also can be produced via RNA and epigenetic modifications in the male germline that are transmitted through the sperm to the zygote (reviewed in Kumar *et al.* 2013). Like SD, zygotic drive is predicted by theory to more readily evolve on the sex chromosomes because all or part of the X and Y (or W and Z) are nonrecombining but also because there is covariation between sexually dimorphic phenotypes and the presence or absence of the sex-linked zygotic driver in offspring that facilitates evolution via “green-beard effects” (Miller *et al.* 2006; Rice *et al.* 2008, 2009).

The empirical verification of potential cases of SA-ZD differs importantly from that of the two most common causes of sex ratio distortion (SD and SK) because SA-ZD has no simple diagnostic phenotype(s). SK is unambiguously identified by the matrilineal—but not patrilineal—transmission of the female-biased sex ratio phenotype, coupled with the elimination of this phenotype by treatment with antibiotics. SD is strongly indicated when total mortality within families is insufficient—when applied to the rarer sex—to account for the observed magnitude of the sex ratio bias. In sharp contrast, SA-ZD is intrinsically confounded with genetic variation for sex-specific viability (Rice *et al.* 2008) and it must be disentangled from this and other alternative etiologies leading to sex ratio distortion via a laborious, multistep process-of-elimination (Friberg *et al.* 2011; Friberg and Rice 2014). As a consequence, SA-ZD is predicted to be “hidden in plain sight” because when active in a genome, it would be easily overlooked as being caused instead—and more parsimoniously—by other causes, especially genetic variation for sex-specific viability.

For example, Noor and Coyne (1995) observed a strongly female-biased sex ratio in an inbred line of *D. simulans* (hereafter “SKEW”) that had been constructed by bringing together different recessive markers—from different stocks—located on all of the major chromosomes. Such mixing of genetic variation from different stocks potentially separates drivers from their suppressors and thereby exposes a latent sex ratio distorter. In reciprocal crosses to a line with an even sex ratio (Florida City), the sex ratio bias was observed: i) to be present in F_1_ families when the sire was from the SKEW line but disappear in families from their F_1_ sons and ii) to be absent in F_1_ families when the sire was from the Florida City line but be present in families of F_1_ sons from these sires. This pattern was consistent with an X-linked SD driver, a conclusion supported by the fact that the sex ratio phenotype in the SKEW line was not eliminated by treatment with antibiotics. The causative X-linked factor (named *skew* by Noor and Coyne 1995) was mapped to the proximal end of the X chromosome, but sample sizes were small, and an error-prone protocol was used to classify sex ratio biased *vs.* even families, so this mapping must be considered to be exploratory. When the two reciprocal crosses were repeated and the proportion of unhatched eggs was compared between the crosses, however, there was a nearly perfect match between the extra no-hatch rate in the cross with SKEW sires and the observed magnitude of sex ratio bias in families from these sires, assuming all the extra no-hatch rate was caused by mortality in sons.

Because of the match between level of elevated mortality in families of SKEW sires and the magnitude of sex ratio bias in their offspring, Noor and Coyne (1995) concluded that “the reduction in egg hatch rate could account for the sex ratio bias, and we cannot conclude that the skew [in sex ratio] results from meiotic drive.” This observation, coupled with their findings of i) patrilineal but not matrilineal inheritance of sex ratio distortion in F_1_ offspring and ii) no effect of antibiotic treatment on this phenotype in the SKEW line, resulted in Noor and Coyne terminating their search for the cause of the sex ratio distortion in the SKEW line because it was “associated with increased egg lethality.” But these observations are exactly what would be predicted by the operation of SA-ZD, which had not yet been theoretically described, and they illustrate how easily SA-ZD may have been overlooked when data supported its potential occurrence in previous studies.

Friberg *et al.* (2011) carried out new experiments on the SKEW and Florida City lines, including crosses to compound-X dams, and their results were fully consistent with the operation of SA-ZD; however, because they only measured sex ratio in newly eclosing subadults, they were not able to unequivocally rule out a complex alternative explanation in which the sperm competitive ability of sires changed in dams from different crosses in a manner that exactly matched the observed sex ratio patterns among crosses. In this study, I eliminate this possibility by measuring the sex ratio in both eclosing subadults and embryos and find that the same X-linked sex ratio distorter codes for both SD and SA-ZD, *i.e.*, part of the observed sex ratio distortion in the SKEW line was attributed to each of these processes. I begin by describing a general process-of-elimination screen to disentangle X-linked SA-ZD from other potential factors leading to sex ratio distortion. This screen should be applicable to a wide diversity of model and nonmodel organisms.

## A process-of-elimination screen for SA-ZD

Here I describe a general screen to disentangle X-linked SA-ZD from SK, X-linked SD, and/or male-specific viability genes—that individually or collectively produce the observed sex ratio distortion in a line of interest. The less common alternative causes of female-biased sex ratios (CYTO-ASEX, CYTO-FEM, and X- and Y-FEM) will be considered separately at the end of this section. Because SA-ZD does not have an unambiguous diagnostic phenotype, it is detected by a process-of-elimination, *i.e.*, by showing that at least some of the sex ratio distortion observed in a line with %♀ > 50 is not due to other candidate processes.

The screen begins with the observation that %**♀** > 50 in a line, and we set out to test whether the female-bias is at least in part caused by X-linked SA-ZD. To demonstrate SA-ZD, it must be shown that: i) At least part of the %**♀** > 50 phenotype is due to SK rather than sperm-killing alone; ii) X_SR_ is required in fathers to kill sons but only via a paternal effect rather than being inherited from the mother and expressed in the sons themselves; and iii) It does not matter from where the Y, autosomes, or cytoplasm/mitochondria come (SR or EVEN), since they are not involved in the killing.

**Figure 1:**
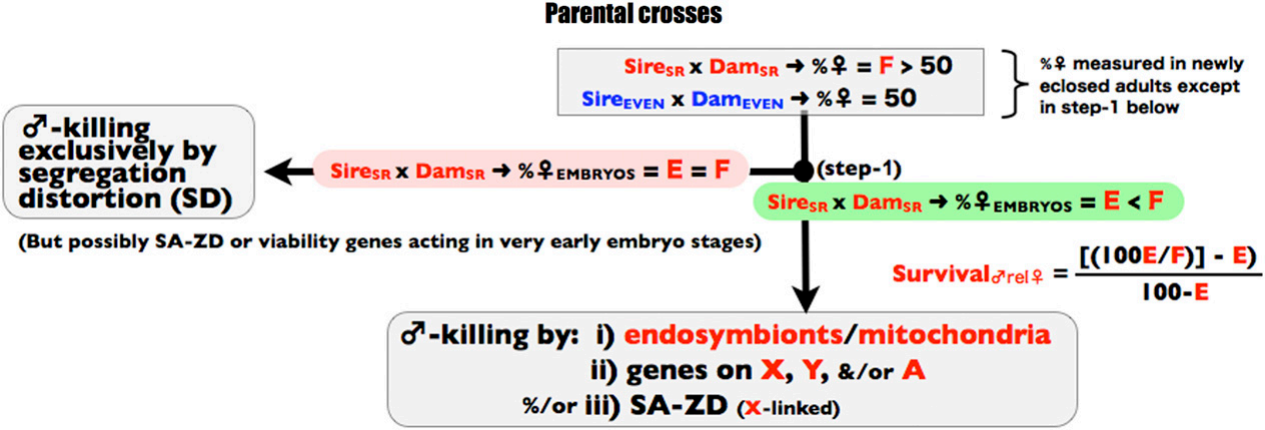
Step 1 of the process-of elimination assay. Red denotes genes, individuals, or parameters from the SR line and correspondingly blue for the control line. In this figure, and all subsequent figures, the symbols “E” and “F” refer to the %♀ in the cross SR × SR.

**Figure 2:**
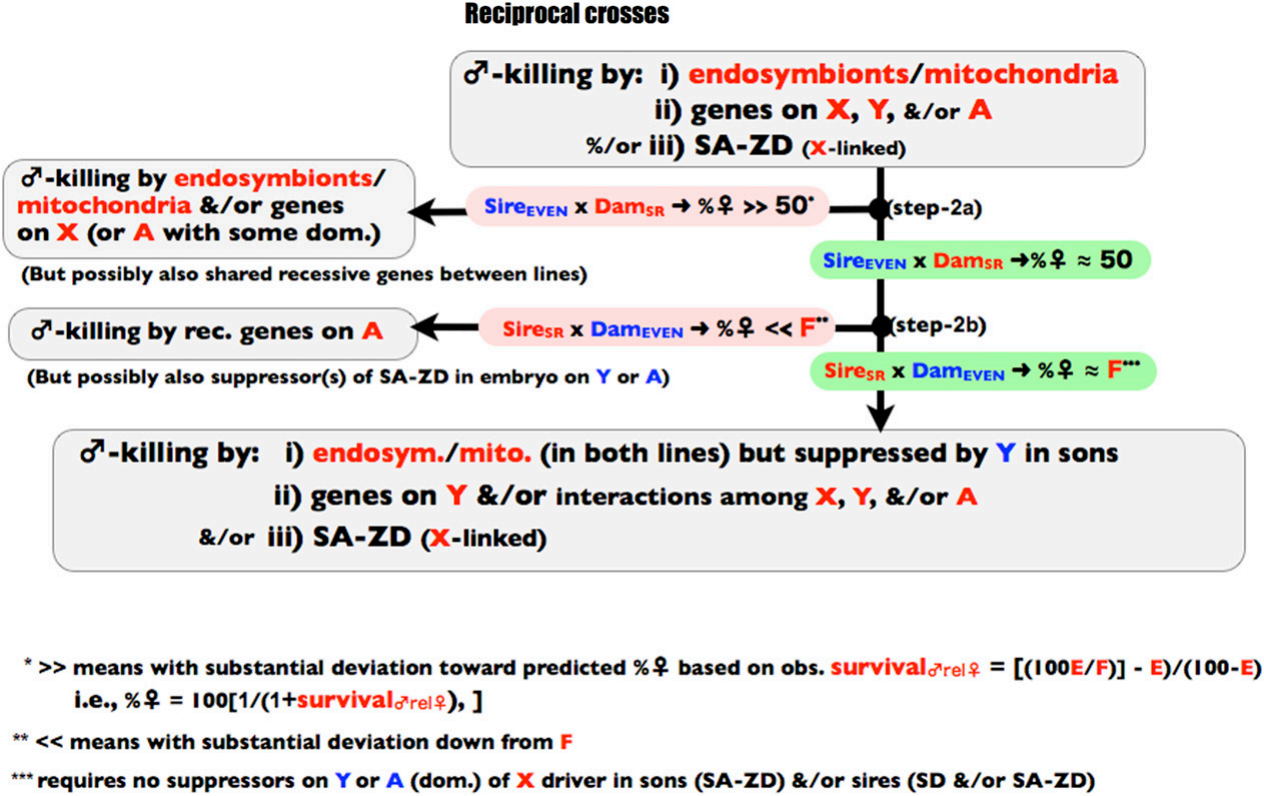
Step 2 of the process-of elimination assay. Red denotes genes, individuals, or parameters from the SR line and correspondingly blue for the control line.

**Figure 3:**
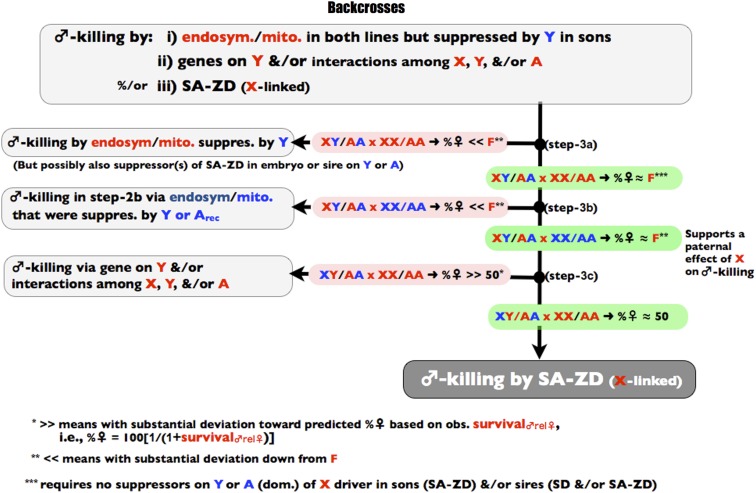
Step 3 of the process-of elimination assay. Red denotes genes, individuals, or parameters from the SR line and correspondingly blue for the control line.

**Figure 4:**
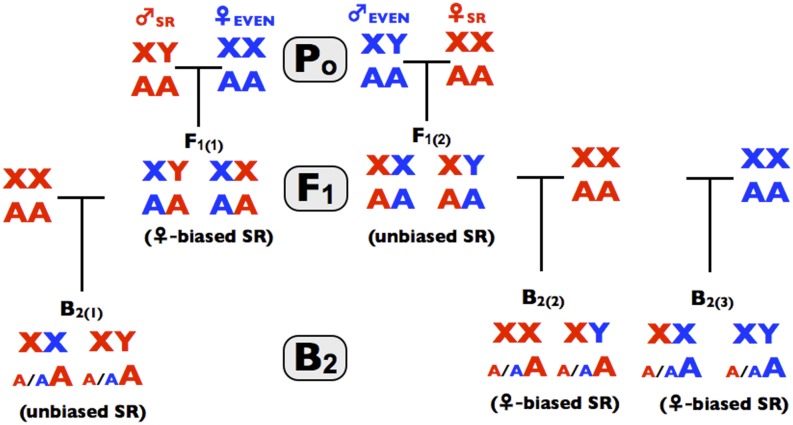
The crosses use in the process-of-elimination assay. Red denotes genes, or individuals from the SR line and correspondingly blue for the control line. P_o_ denotes the parental crosses in step 1 of the process-of elimination assay, F_1_ denotes progeny from the parental crosses (step 2 of the assay), and B_1_ denotes progeny form the backcrosses (step 3 of the assay). Parenthetical text denotes the observed pattern found in this study.

The three stages of the screen are depicted in Figure 1, Figure 2, and Figure 3 and all of the crosses used in the screen are summarized in Figure 4. The screen is designed to operate in a manner analogous to a dichotomous taxonomic key. At the top of Figure 1, an observed female-biased sex ratio is potentially due to X-linked SD, endosymbiont/mitochondrial-coded SK, X-linked SA-ZD, and/or nuclear viability factors that reduce male survival relative to female survival. As one proceeds down the binary decision key, any step to the left terminates the screen and leads to the conclusion that the available data are consistent with sex ratio distortion being caused by one or more factors that do not include SA-ZD. Navigating to the left does not unambiguously identify the true cause(s) of sex ratio distortion—it only demonstrates that there is insufficient evidence to conclude that SA-ZD is operating. If one navigates to the base of the key in Figure 4, then the step-wise process-of-elimination demonstrates that at least some of the observed sex ratio distortion in the line is due to SA-ZD. This screen will fail to detect SA-ZD (false negatives) when it is weak and/or when its suppressors are present in the control line with an even sex ratio, but it should not lead to false positives –as described more fully in the discussion section.

A critical first step in the screen is the sexing of early-stage embryos—a time point before all or most ontogenetic, male-specific mortality factors have had an opportunity to act. Such embryo-sexing typically will require the use of techniques relying on polymerase chain reaction applied to DNA extracted from individual embryos or staining embryos with fluorescence-labeled antibodies or oligonucleotides that bind sex-specific molecular markers. These techniques, however, can currently be used on a wide diversity of species, and the expanding availability of sex-specific antibodies, X- and Y-linked genetic markers, and genome-wide sequences of nonmodel organisms, will make the applicability of the screen expand with time. Because the sexing of embryos can be time-consuming and expensive, the screen for SA-ZD minimizes the number of times that sexing embryos must be done, *i.e.*, it need only be done in the first step of the 6-step screen, and only on the line suspected of harboring SA-ZD.

## Parental crosses

The screen begins with two lines. A target line (classified as “SR” and shown in red in the figures) that produces a female-biased sex ratio at birth, fledging, eclosion, or some other early life history stage preceding sexual maturity and the associated potential onset of strong differences in sex-specific morality due to different behaviors, ecologies, and life histories. For simplicity, I hereafter categorize all post-embryo/pre-sexually mature stages as “subadults”. The control line (classified as “EVEN” and depicted in blue in the figures) has a 50%♀ subadult sex ratio (or a value close to 50%, because large sample sizes commonly detect small sex ratio deviations in inbred lines, presumably as the result of minor differences in sex-specific viability). Sex ratio should be measured under benign conditions to minimize any contribution to subadult sex ratio from sex-specific viability effects.

## Step 1: sexing embryos

The first step in the screen is to sex a sample of subadults and embryos from the SR line (Figure 1, step 1). Ideally the sexing of embryos should be done at or close to syngamy to ensure that any sex-specific postzygotic mortality is detected by the comparison of embryonic *vs.* subadult sex ratios; however, sexing such early-stage embryos would usually require fluorescence in situ hybridization of X- and Y-specific genetic markers (typically satellite DNA, *e.g.*, see Ferree and Barbash 2009), which is impractical in many applications. When embryos are sexed at a later stage, a false-negative for sex-specific mortality due to SK, SA-ZD, and/or viability genes can occur. Nonetheless, known cases of sex-specific mortality from SK-inducing endosymbionts (*i.e.*, *Wolbachia* and *Spiroplasma*) would be detected by sexing fertilized eggs at more advanced stages of embryonic development (*e.g*., see Kageyama *et al.* 2009; Riparbelli *et al.* 2012). If the sex ratio bias of embryos from the SR line is the same (or nearly so) as that of subadults (Figure 1, step 1), then the sex ratio distortion is plausibly due to X-linked SD exclusively (or nearly so). If there is a nontrivial increase in the degree of female bias in subadults compared with embryos, however, then some additional sex ratio−distorting mechanism(s) is operating (assumed here to be higher male mortality compared with females, but feminization of genetic males is covered at the end of this section) and the possible contribution of SA-ZD can be determined by continuing the process-of-elimination. In this case, the reduced male survival (compared with females and occurring after the time point when embryo sex ratio was measured) can be calculated by: survival_♂rel♀_ = [100*E/F − E]/[100 − E)], where E is the %♀ in embryos from the cross SR × SR, F is the %♀ in subadults in the cross SR × SR, and survival_♂rel♀_ is measured as a proportion between zero and one. Note that the symbols “E” and “F” refer specifically to the %♀ in the SR x SR cross, and their values will be used as benchmarks in later parts of the screen.

## Step 2: reciprocal crosses

The next step is to carry out reciprocal crosses between the SR and EVEN lines. If the %♀ from the cross Sire_EVEN_ × Dam_SR_ >> 50% (see Figure 2, step 2a), then the elevated male mortality (hereafter “male-killing”) is feasibly caused by SK (coded by endosymbionts or mitochondria), one or more X-linked genes, and/or one or more genes on the autosomes with a nontrivial level of dominance (or shared recessive alleles between lines). The “>>” inequality implies substantial deviation, *i.e.*, most of the deviation toward the predicted %♀ based on the observed survival_♂rel♀_ in step-1, *i.e.*, %♀ = 100[1/(1+survival_♂rel♀_)]. If this inequality is met, then SA-ZD cannot be unambiguously deduced from the available data. If this inequality is not met, then the sex ratio in the reciprocal cross is evaluated (step 2b). If the %♀ from the cross Sire_SR_ × Dam_EVEN_ << F, then there is evidence for male-killing due to one or more recessive (or nearly recessive) genes on the autosomes. In this case the inequality “<<” symbol means that most of the proportional deviation down from F is observed compared with the total possible deviation of F − 50. A false-negative for SA-ZD could occur at this step if the EVEN line carried suppressors (on the Y or autosomes) of the SA-ZD agent operating in the SR line that rescues sons from paternal-effect mortality. Such suppressors in the EVEN line are not expected but might nonetheless be present due to fortuitous pleiotropy or as remnants of a previously active drive suppressor system.

If both inequalities in steps 2a and 2b are not met, then by the expanded process-of-elimination the sex ratio bias in the SR line is due to (at least in part): i) SK from endosymbionts that is suppressed by Y_EVEN_ in sons; ii) male-killing genes on Y_SR_ or due to epistatic interactions among Y_SR_, X_SR_, and/or A_SR_; and/or iii) X-linked SA-ZD (Figure 2, bottom). Passage through step 2b could also occur, however, if there was latent SK via endosymbionts that are unique to the EVEN line and that were suppressed in sons by Y_EVEN_ and/or a recessive genes(s) in the EVEN line –a possibility that must be ruled out in step 3b below.

## Step 3: backcrosses

To extend the process-of-elimination further, F_1_ males from the reciprocal crosses are backcrossed to dams from the SR and EVEN lines (see Figure 3 and Figure 4). If in step 3a of Figure 3 the %♀ from the backcross X_SR_Y_EVEN_/A_SR_A_EVEN_ × X_SR_X_SR_/A_SR_A_SR_ is << F, then there is evidence that step 2a missed the action of SK (coded by endosymbionts and/or the mitochondria in the SR line) because male-killing was suppressed in sons by Y_EVEN_. This result leads to the conclusion that there is insufficient evidence to support the operation of SA-ZD. If in step-3b of Figure 3 the %♀ from the backcross of these same sires to the EVEN dams (X_SR_Y_EVEN_/A_SR_A_EVEN_ × X_EVEN_X_EVEN_/A_EVEN_A_EVEN_) is <<F, then one may have passed through step 2b because there was a latent sex ratio distortion in the EVEN line coded by mitochondria/endosymbionts (different from those in the SR line) that was suppressed by Y_EVEN_ or recessive suppressors on the autosomes. Finding %♀ ≈ F in step 3b of Figure 3 rules out this possibility and further corroborates the prediction of an X-linked paternal effect mediating SA-ZD. If in step 3c of Figure 3 the %♀ from the reciprocal backcross X_EVEN_Y_SR_/A_SR_A_EVEN_ × X_SR_X_SR_/A_SR_A_SR_ is >> 50, then there is evidence for male-killing coded by Y_SR_ or due to epistatic interactions among Y_SR_, X_SR_, and A_SR_. This result leads to the conclusion that there is insufficient evidence to conclude the operation of SA-ZD. If none of the inequalities are met in steps 3a,b,c (and previously in steps 2a and 2b of Figure 2), then by a process-of-elimination, SD, SK, and/or male-specific viability genes cannot fully account for the sex ratio bias, and one can conclude that SA-ZD contributes to the observed sex ratio bias in the SR line.

Lastly, I consider the four less-common causes of sex ratio distortion, *i.e.*, CYTO-ASEX, CYTO-FEM, X-FEM, and Y-FEM. CYTO-ASEX will produce the same pattern as SD in the process-of-elimination assay (*i.e.*, a biased sex ratio in early-stage embryos that is equivalent to that seen in subadults), so navigating to the left in step 1 (*i.e.*, to ♂-killing exclusively by SD) will also include sex ratio distortion due to this processes. CYTO-FEM, X-FEM, and Y-FEM will also cause one to navigate to the left in step 1, so long as sex is measured with sex-specific molecular markers in sufficiently aged embryos (like antibodies to the sex-specific protein coded by the Sxl gene in *Drosophila* embryos after the cellularization stage) and not based on the sex chromosome karyotype. If sex in embryos is measured by the sex chromosome karyotype or at a stage too young to detect the reversal of genetic sex, feminization will nonetheless still be distinguished from SA-ZD. In this case, CYTO-FEM will produce the same pattern as SK in the process-of-elimination assay (*i.e.*, exclusively matrilineal transmission of the female-biased sex ratio phenotype), so navigating to the left in steps 2a, 3a, or 3b (*i.e.*, to ♂-killing by endosymbionts) also will include sex ratio distortion due to CYTO-FEM. Finally, X-FEM and Y-FEM, if not detected in step 1, will produce the same pattern as a male-killing gene on the X or Y chromosomes, respectively, so navigating to the left in steps 2a or 3c (*i.e.*, to ♂-killing by the X, Y and/or interactions among the X, Y, and/or A) will also include sex reversal by the sex chromosomes.

## Materials and Methods

### Stocks

The SKEW and Florida City (= EVEN) stocks used in this study were obtained from the UC San Diego Drosophila Species Stock Center: SKEW = 14021-0251.093 Dsim\g[1]; cn[1]; e[1], skew[1], and Florida City = 14021-0251.165 Dsim\wild-type. The SR line used in the process-of-elimination screen to test for SA-ZD was produced by crossing SKEW males to EVEN females and then making recombinant inbred lines (RILs) by repeated brother/sister matings for at least 15 generations before being used in process-of-elimination assay. Six of these RILs were used to create stocks that were propagated by crossing two males to four females each generation. Each of these stocks had a sex ratio of approximately 85% females, and one of these (RIL-6) was used as the “SR” line in process-of-elimination screen for SA-ZD.

The X chromosome from the SR line also was recombined into a compound-X line [lz[sp]/Y males and C(1)RM, y, w /Y females] by repeated backcrossing for 25 generations before use here. The compound X line was kindly supplied by Yun Tao. An X chromosome containing the Paris SD driver (provided by Yun Tao) was also recombined into the C(1) line for 25 generations before use so that the SR X chromosome and an X containing the Paris SD driver were available for comparison in the same genetic background, *i.e.*, that of the C(1) line. Paris and SR males from these compound-X lines will be referred to as ♂-Paris_C(1)_ and ♂-SR_C(1)_. In the assay for the suppression of the Winters and Durham SD drivers described below, a stock [UC San Diego Drosophila Species Stock Center 14021-0251.194 Dsim\wild-type = (Begun) sim6, hereafter sim6] was used.

### Measuring sex ratio in embryos

Males and females were 3−6 d old when used and all culturing was done at 25°. Groups of 25 males and 25 females were placed into each of 12 vials with the top of the cornmeal-molasses-yeast food medium seeded with live yeast. Flies were transferred to fresh vials at 8:00, 13:00, 18:00, and 22:00. The vials from 18:00 were incubated at ~25° until 6:00 the next day, at which time eggs were collected from the surface by washing with distilled water into an egg-collection basket with a nylon mesh bottom. The age of these eggs was 8- to 12-hr postegg deposition. Residual yeast and fly medium were rinsed from the eggs while in the collection basket with a gentle stream of DI water. To dechorionate the eggs, the egg-collection basket (with the eggs on the nylon mesh bottom) was next immersed in a small beaker containing a freshly made solution of 50% commercial-grade chlorine bleach (4 mL of bleach diluted to 8 mL with deionized water) until half the egg-collection basket was submerged. The bleach and eggs were continuously mixed by repeatedly pipetting bleach from the outside of the basket into the interior of the basket. After 2 min, the egg-collection basket was removed from the bleach, and residual bleach was diluted away by pipetting a stream PBST (*i.e.*, phosphate-buffered saline solution with Triton-X detergent added to prevent eggs from clumping) over the dechorionated eggs. The eggs were next transferred to a 1-mL vial by washing them off the nylon mesh bottom of the egg basket with a pipetted stream of heptane (1 mL). Next, 1 mL of methanol was added, the vial was capped, and was then shaken for 30 sec to devitellinate and fix the embryos. Embryos were next washed twice by pipetting off the fluid above the settled embryos, adding 500 µL of fresh methanol, capping, and gently inverting the vial and then letting the embryos settle to the bottom for 10 min. Embryos were next gradually rehydrated into PBST and stained with antibodies.

Embryos were stained with mouse anti-Sxl (Developmental Studies Hybridoma Bank, University of Iowa) primary antibody and rabbit anti mouse IgG FITC (Invitrogen) secondary antibody. To begin the staining protocol, most of the PBST was pipetted off settled embryos that had been transferred to a 0.5-mL microcentrifuge tube. Sufficient primary antibody (10-fold diluted) was added to double the volume of the settled mass of embryos followed by a rocking motion for 30 min and then incubated overnight without rocking at 4°. The next morning, the primary antibody solution was pipetted off the settled mass of embryos and then the embryos were rinsed six times by adding 500 μL of PBST, allowing the embryos to sink to the bottom and then removing supernatant. The embryos were next washed six times by adding 500 μL of PBST, gently agitating them in the PBST for 10 min, allowing the embryos to settle to the bottom, and then removing the supernatant.

All of the following steps were performed in a dark room at low illumination. Secondary antibody (200-fold diluted) was added, using a sufficient volume to double the volume of the mass of settled embryos. This mixture was incubated at room temperature (~25°) for 4 hr with continuous gentle shaking. The secondary antibody was next removed, and the embryos were rinsed and washed as described previously for the primary antibody. After the last wash, the PBST overlying the settled embryos was replaced with 60 µL of Fluoroshield (with 4′,6-diamidino-2-phenylindole), gently rocked to suspend the embryos in the Fluoroshield, and then the embryos were allowed to sink for 10 min at room temperature. With the use of a yellow pipette with the tip cut off, 40 µL of the embryo/Fluoroshield solution was taken from bottom of the tube, placed on a microscope slide, covered with a coverslip, and sealed with clear nail polish. Embryos were immediately viewed and photographed with a microscope equipped with epifluorescence. In each nonoverlapping microscope field, all embryos (male and female) were first counted using DAPI illumination and filter set. Female embryos (that were stained by the ant-Sxl and fluoresced a bright green) were next counted using FITC illumination and filter set (male embryos were dark in this context).

### Measuring sex ratio in newly eclosed subadults

Subadult sex ratio was measured on eggs collected from the same flies used to produce the eggs used to sex embryos. Flies were transferred to fresh vials daily at 8:00, 13:00, 18:00, and 22:00. Vials from the 18:00 transfer were used to measure embryo sex ratio. Vials from the transfers immediately before (13:00) and after (22:00) this sample were used to measure subadult sex ratio. Excess eggs were manually culled from the surface of each vial, so that no more than ~150 remained before the eggs hatched. This was done to minimize variation in larval density during development and prevent overcrowding. Subadults were collected on from each vial daily until they were 8 d after eclosion of the first subadults to count all offspring but not include any grand-offspring emerging in the next generation. To maintain consistency, subadult sex ratios were measured in the same way and at the same times in all three generations of the process-of-elimination screen (Figure 4).

### Test for suppression of the Winters and Durham drivers

Tao *et al.* (2007) showed that the Winters SD driver is suppressed by the dominant allele Nmy and that the Durham SD driver is suppressed by the dominant allele Tmy. Kingan *et al.* (2010) used sequencing data to show that the line sim6 was homozygous for the gene Nmy, and crossing results by Yun Tao (personal communication) indicate that this line is also homozygous for Tmy. To test whether the SR driver is suppressed by the dominant suppressors of the Winters and Durham drivers, I crossed SR males to females from the sim6 line. Sons from this cross should carry the SR driver but be heterozygous for the dominant suppressors of both the Winters and Durham SD drivers. If these sons have a sex ratio distortion phenotype, then this result would support the conclusion that SR is not one of these two drivers.

### Test for suppression of the Paris driver

All crosses in this section are summarized in Supporting Information, Figure S1. Tao *et al.* (2007) showed that a Y chromosome from *D. sechellia* suppressed the Paris SD driver. I tested to see whether a Y from *D. sechellia* also suppressed the SR driver. The *D. simulans* stock carrying the Y chromosome from *D. sechellia* that was tested by Tao *et al.* (2007) had been lost from the stock center so a different Y chromosome from *D. sechellia* was used (kindly provided by Michael Shahandeh from the stock SynA, which I will refer to as Y_sec_). I first recombined Y_sec_ into the C(1)Y_sim_ females by crossing males from the SynA line of *D. sechellia* (X_sec_Y_sec_) to C(1)Y_sim_ females of D. simulans. C(1)Y_sec_ daughters were next mated to males from the SR line to produce sons that were X_SR_Y_sec_ and these sons were backcrossed to SR females eight additional times and then the sex ratio of their broods was measured.

To directly compare the ability of Y_sec_ to suppress the sex ratio phenotype of X_SR_Y_sec_ and X_Paris_Y_sec_ males, I next crossed X_SR_Y_sec_ males (from the backcross generation-3 in which Y_sec_ was being backcrossed into the SR line) to C(1)Y_sim_ females to produce C(1)Y_sec_ daughters. These were then crossed to ♂-SR_C(1)_ and ♂-Paris_C(1)_ sires. Sons from this cross were X_Paris_Y_sec_ or X_SR_Y_sec_ and had identical autosomal genetic backgrounds and maternal effects. These sons were used as sires and mated (100 matings of 1♂ × 2♀ for each type of sire) to EVEN dams and the sex ratio of their families was measured. These crosses measure the sex ratio phenotype of X_SR_ and X_Paris_ chromosomes in sires when there is potential suppression by Y_sec_. For comparison, ♂-SR_C(1)_ and ♂-Paris_C(1)_ sires (which have identical maternal effects and genetic backgrounds except for the X chromosomes) also were mated to EVEN females (100 matings of 1♂ × 2♀ for each type of sire), and the sex ratio of their families was measured. These crosses measure the sex ratio phenotype of X_SR_ and X_Paris_ chromosomes in sires when there is no potential suppression by Y_sec_. If both X_Paris_Y_sim_ and X_SR_Y_sim_ sires produce broods with ♀-biased sex ratios and neither X_Paris_Y_sec_ nor X_SR_Y_sec_ sires produce broods with ♀-biased sex ratios, then Y_sec_ fully suppresses both drivers and there is evidence that both are the Paris driver.

### Test for recombination between the Paris and SR drivers

All crosses in this section are summarized in Figure S2. To measure the recombinational distance between the Paris and SR drivers, in generation 0 (G_0_) Paris males (♂-Paris_C(1)_) taken from the compound-X line were mated to females from the SR line (cross 1 in Figure S2). G_1_ daughter therefore had the Paris driver on one X chromosome and the SR driver on the other X. Progeny sons (from G_1_ × G_1_ → G_2_ progeny sons) would carry SR or Paris alone if no recombination occurred between the drivers and both SR and Paris or neither driver if recombination did occur between them. Therefore 400 G_2_ sons were crossed to EVEN dams (1 sire × 2 dams), and the sex ratio of their families was measured: twice the proportion of sons producing broods with ~50%♀ estimates the recombinational distance between the drivers. Some sires, however, may produce families with nonsignificant ♀-bias because of low statistical power associated with small family size and/or because the Paris driver is known to have incomplete penetrance, *i.e.*, it sometimes fails to produce sex ratio bias when present (Montchamp-Moreau *et al.* 2001). For these reasons, any sons that did not produce ♀-biased families might be a false-negative for the presence of a driver (Paris or SR) on their X chromosome. To test for this possibilities, daughters from all G_2_ sires that failed to produce statistically significant ♀-biased families (that had crossed to their brothers prior to progeny counting in the sex ratio assay) were used to produce grandsons from each sire (cross 4 in Figure S2). The sex ratio of families produced by 10 of these grandsons (producing > 60 offspring) was measured when they were mated to EVEN dams (1♂ x 2♀, cross-5 in Figure S2). Only half of the sons at most would be expected to carry a sex ratio distorter (SR or Paris) because their mother was at most heterozygous for an undetected driver. But with 10 sons screened, the probability that at least one of the 10 sons will carry the driver if she were a heterozygote is 1−1/2^10^ > 0.999. Therefore, this grandson assay should detect with high probability any sires in the G_2_ that failed to produce ♀-biased sex ratios due to small family size and/or lack of expression of the Paris driver when present.

### Comparing mortality and sex ratio in the SR line

To determine whether all the sex ratio bias in the SR line could be attributed to greater mortality of males, I crossed one male to one virgin female and housed replicate pairs in separate vials with small amounts of live yeast applied to the killed-yeast medium. After 2 d, I checked the vials for the presence of larvae and discarded all of those not containing larvae. The remaining vials (with fertile males and females) were individually transferred to fresh vials containing a small puddle of yeast suspension that had dried to the surface the previous evening, producing a thin veneer of live yeast about a half centimeter in diameter on the surface of the food medium. Nearly all eggs were laid within this yeast-covered location and they were easily counted the morning after they were deposited. Adult flies were discarded 12 hr later; the eggs were counted and then compared to the numbers of emerging subadults to measure subadults/egg.

### Statistical analysis

All %♀ estimates and their 95% confidence intervals were obtained using the JMP statistical software package with the generalized linear model platform assuming beta-binomial error terms. Vials containing male and female offspring were used as the unit of replication.

## Results

The SKEW line that was originally studied by Noor and Coyne (1995) and their control line with a 50:50 sex ratio (Florida City, hereafter referred to as EVEN to match the labeling in Figure 1, Figure 2, Figure 3, and Figure 4) were obtained from the Drosophila Species Stock Center and scored for sex ratio. In newly eclosing flies, the sex ratio for the SKEW line was 57.3%♀ with a 95% CI of (55.4, 59.2) and that for the EVEN line was 50.3%♀ with a 95% CI of (48.5, 52.0). The low sex ratio bias of the SKEW line (down from 70% ♀ when Noor and Coyne first started their work, but similar to the 61.3%♀ that they reported near the end of their 3-year study) and difficulty keeping the inbred skew stock alive because of low viability, fertility, and egg-hatch rate, prompted me to make RILs via repeated generations of brother-sister matings from an initial cross between SKEW males × EVEN females. Some of these lines produced strong sex ratio bias (> 90%♀) but the lines with the strongest %♀ were invariably lost because they eventually produced 100%♀. Five RILs eventually were established with consistent sex ratios of about 85%♀, and one of these (RIL-6, hereafter SR to match the labeling in Figure 1, Figure 2, Figure 3, and Figure 4) was used with the EVEN line to screen for the operation of SA-ZD.

To begin the screen for SA-ZD, I compared the sex ratio of embryos and subadults in the SR line. The sex ratio in a sample of 1423 embryos was %♀ = E = 76.5 with a 95% CI of (74.0, 78.7). The sex ratio in a sample of 6588 newly eclosing subadults (produced by the same crosses that produced the embryos, and on the same day) was %♀ = F = 83.9 with a 95% CI of (82.7, 84.6). The finding of F > E clearly demonstrates that greater mortality of males relative to their sisters—or feminization later in development—contributed to part of the observed sex ratio bias in the SR line. The feminization alternative is eliminated in later steps of this process-of-elimination screen. As a control, I also scored the sex ratio of embryos and subadults in the EVEN line: Embryos: %♀ = E_FC_ = 49.3 with 95% CI of (45.8, 52.7) and N = 1009; Newly eclosing subadults: %♀ = F_FC_ = 50.2 with 95% CI of (48.5, 52.1) and N = 2821.

The strongly distorted sex ratio in embryos (E = 76.5%♀) provides evidence that male-specific mortality is not the sole contributor to the observed sex ratio distortion in the SR line, unless a substantial proportion of males die during very early ontogeny—at a stage before the embryos were screened. To further test the hypothesis of more than one phenotype contributing to the sex ratio distortion, I measured the total egg-to-subadult mortality in another sample of eggs produced by SR females mated to fertile SR males (total eggs screened = 1502). The ratio of subadults per egg was estimated (by the average number of eclosing subadults per egg) to be 84.0% with a 95% CI of (80.3, 87.5), so I can be 95% confident that total mortality was no greater than 20%. If the sex ratio at syngamy was 50%♀ and all this 20% mortality went to males, the subadult sex ratio would be 100[0.5/(0.5+0.3)] = 62%♀, far less than the 83.9%♀ observed in subadults (and its 82.73% 95% lower bound) and embryos (with a 74.0% 95% lower bound), so clearly some factor besides higher mortality of males relative to their sisters contributed to the sex ratio distortion in the SR line. SD is a feasible candidate factor but other possibilities include partial asexual reproduction by SR females (CYTO-ASEX), and feminization of genetic XY males (CYTO-FEM, X-FEM, and/or Y-FEM). Because sex-reversed XY embryos would be expected to lead to sterile females (Casper and Van Doren 2009)—and the SR line had low rates of female infertility (data not shown)—CYTO-FEM, X-FEM, and/or Y-FEM are not feasible candidates. Because “the occurrence of natural parthenogenetic development is extremely low in ... Drosophila simulans” (Markow 2013), CYTO-ASEX is also not a feasible candidate, leaving SD as the only strong candidate factor causing the sex ratio distortion observed at the embryo stage. This conclusion is corroborated below where I report zero recombination between the sex ratio driver in the SR line and an established segregation distorter in heterozygous females, and the complete elimination of the sex ratio phenotype in the SR line by a Y chromosome that suppresses this distorter.

The estimated extra postembryonic mortality of males compared to their sisters was calculated to be 37.5%, *i.e.*, 100 − %survival_♂rel♀_ = 100 − 100[100*E/F − E]/[100 − E)]. By comparison, the estimated extra mortality/incapacitation of Y bearing sperm relative to X-bearing sperm was estimated to be 69.3%, *i.e.*, 100 − %survival_YrelX_ = 100 − 100[100*50/E) − 50]/[100 − 50)]). These measures indicate that the level of postembryonic mortality of males relative to death/incapacitation of Y-bearing sperm is estimated to be 37.5/69.3 = 0.54, or about half as strong a mortality agent as SD in its contribution to total sex ratio distortion.

In step-2 of the screen for SA-ZD, I carried out the reciprocal crosses between the SR and EVEN lines. In the cross Sire_EVEN_ × Dam_SR_ (step 2a, Figure 2), I found the %♀ ≈ 50% and not >> 50% (%♀ = 52.7% with 95% of CI [51.1, 54.4], N = 7013). In the cross Sire_SR_ × Dam_EVEN_ (step 2b, Figure 2) I found the %♀ to be ≈ F and not <<F (%♀ = 84.6% with 95% of CI [82.9, 86.2], N = 5434). Collectively these two results are consistent with male killing via SA-ZD but inconsistent with i) SK unless it is suppressed by Y_EVEN_ in sons and ii) SK nuclear genes, unless they are Y_SR_-linked and/or include epistatic interactions among X_SR_, Y_SR_ and/or Y_SR_ (Figure 2, bottom).

In step 3, backcrosses were done with F_1_ males sampled from the two reciprocal crosses from step-2. In the first backcross, X_SR_Y_EVEN_/A_SR_A_EVEN_ × X_SR_X_SR_/A_SR_A_SR_ (Figure 3, step 3a), the %♀ was ≈ F and not << F (%♀ = 87.3%, with a 95% CI of 85.6,88.3 and N = 4307). This finding is inconsistent with the possibility (in step 2a of Figure 2) of SK that was suppressed by Y_EVEN_ in sons. In the backcross of X_SR_Y_EVEN_/A_SR_A_EVEN_ × X_EVEN_X_EVEN_/A_EVEN_A_EVEN_ (step 3b in Figure 3), the %♀ ≈ F (%♀ = 87.2%, with a 95% CI of [85.6, 88.7] and N = 4,692). This finding rules out the possibility of a latent and unique sex ratio distorter in the EVEN line that was uncovered by separating it from its suppressors on Y_EVEN_ and/or A_EVEN_. It also supports a paternal-effect of X_SR_ on SK. In the second backcross, X_EVEN_Y_SR_/A_SR_A_EVEN_ × X_SR_X_SR_/A_SR_A_SR_ (Figure 3, step 3c), the %♀ was ≈ 50 and not >> 50 (%♀ = 53.2%, with a 95% CI of (52.1, 54.3) and N = 4,692). This finding is inconsistent with the possibility of any substantive male-killing by nuclear genes that are Y_SR_-linked and/or those that include epistatic interactions among X_SR_, Y_SR_, and/or Y_SR_ sons. In both steps 3a and 3b, the sex ratio was slightly greater than that found in the SR line (%♀ =87 *vs.* %♀ = 84). In both cases the Y chromosome in the sire was Y_EVEN_. Additional experiments (data not shown) indicated that Y_SR_ suppresses sex ratio distortion compared with Y_EVEN_ by a few percent.

In summary, by passing all of the six dichotomous steps in the screen for SA-ZD, a process-of-elimination strongly supports the operation of SA-ZD in the SR line; however, SA-ZD alone cannot account for all of the sex ratio distortion in the SR line, and there is clear evidence for the simultaneous operation of X-linked SD.

Because I passed through steps 1, 2a, 3a, and 3b, I can also conclude that the SA-ZD phenotype was operating and not confused with CYTO-ASEX, CYTO-FEM X-FEM, and Y-FEM, as described more fully in the last paragraph of the previous section describing the rationale for the process-of-elimination screen.

## Is SR a Previously Established Sex Ratio Distorter?

The fact that the SR line exhibits a phenotype consistent with the operation of both X-linked SD and SA-ZD motivates the hypothesis that SD and SA-ZD may have the same source in the SR line. To test whether the SD component of the SR line comes from a know SD driver (*i.e.*, Paris, Durham, or Winters), I carried out a series of experiments.

### Suppressors of the Winters and Durham SD drivers do not suppress sex ratio distortion in the SR line

I first crossed SR males to females from inbred line sim6 (a.k.a. line14021-0251.194 of the Species Drosophila stock center, D. Begun, personal communication) from the collection of wild-derived inbred lines of *D. simulans* that were originally collected by David Begun from an orchard in Winters CA in 1995. This line is homozygous for the dominant suppressor (Nmy) of the Winters SD element (Kingan *et al.* 2010) and genetic crosses (Y. Tao, personal communication) demonstrate that it is also fixed for the dominant suppressor (Tmy) of the Durham SD driver. Sons from this cross produced strong sex ratio distortion (%♀ = 80.5, 95% CI = [78.5, 82.4], N = 4208 offspring screened). The fact that dominant suppressors of both the Winters and Durhan SD drivers failed to suppress the SR driver supports the conclusion (but does not prove) that it is neither of these established drivers.

#### A suppressor of the Paris SD driver does suppress sex ratio distortion in the SR line:

Next I tested to see whether a suppressor of the Paris driver would suppress the sex ratio distortion phenotype in the SR line. A previous study had shown that a Y chromosome from *D. sechellia* suppressed the sex ratio distortion produced by the Paris driver (it actually weakly reversed the sex ratio distortion; Tao *et al.* 2007). I obtained a stock of the Paris driver in an attached-X background (kindly supplied by Yun Tao) and I recombined the SR driver (from RIL-6) into this same genetic background (>25 backcross generations). The *D. sechellia* stock containing the Y chromosome that suppressed the Paris driver (Tao *et al.* 2007) was lost from the Drosophila Stock Center, so I backcrossed another Y chromosome from another *D. sechellia* line (the “SynA” line, kindly provided by Michael Shahandeh, hereafter Y_sec_), into the SR line. After eight backcross generations, the SR line with the Y_Sec_ replacement had little or no sex ratio distortion (%F = 49.9, 95% CI is [45.5, 54.3], N = 493 offspring screened) and after nine backcross generations, the same pattern was observed (%F = 50.9, 95% CI is [46.9, 55.0], N = 577 offspring screened), indicating strong suppression of sex ratio distortion in the SR line.

**Figure 5:**
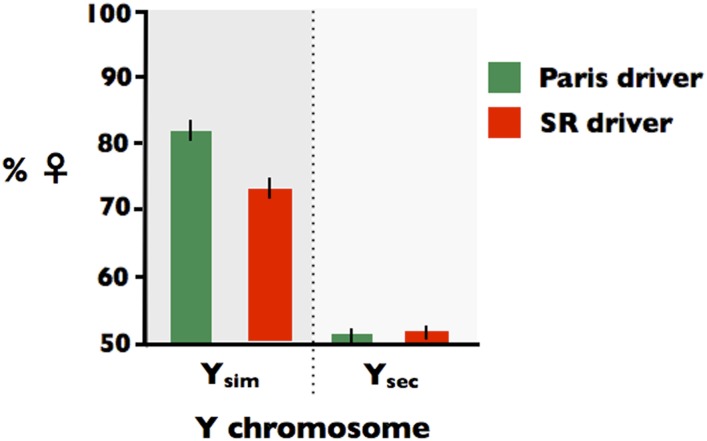
Histogram of the sex ratio of progeny produced by sires expressing the SR driver (red bars) or the Paris driver (green bars) when paired with a Y chromosome from *D. Simulans* (Y_sim_) or *D. sechellia* (Y_sec_).

However, the sex ratio distortion was not reversed as was observed by Tao *et al.* (2007). Because Y chromosomes within *D. simulans* are known to be highly polymorphic for their influence on the level of sex ratio distortion by the Paris SD driver (including sex ratio reversal, see Montchamp-Moreau *et al.* 2001), I next crossed the backcrossed Y_Sec_ males to females from the compound-X stock used to propagate the Paris and SR X chromosomes (X^X_sim_Y_sim_ females; see Figure S1, G_0_). This cross placed the Y_Sec_ into compound-X females (X^X_sim_Y_sec_; see Figure S1, G_1_), from which it could be introduced to males carrying both X_Paris_ and X_SR_ in the same genetic background. Males carrying X_Paris_ or X_SR_ from the attached-X stock were next crossed to the compound-X females carrying Y_sec_ (X^XY_sec_) or females carrying Y_sim_ (X^XY_sim_; see Figure S1, G_2_) and the sex ratios produced by sons from these crosses were measured by mating them to females from the EVEN line (see Figure S1, G_3_). As shown in Figure 5, both X_Paris_ and X_SR_ males carrying Y_sim_ produced biased sex ratios, with the Paris driver producing a larger sex ratio bias (offspring from X_Paris_Y_sim_ sires: %♀ = 82.1 with a 95% CI of [79.7, 84.4] *vs.* offspring from X_SR_Y_sim_ sires: %♀ = 72.0 with a 95% CI of [71.0, 75.0]). When paired with the Y_sec_, however, neither driver produced any substantive sex ratio bias (offspring from X_Paris_Y_sec_ sires: %♀ = 51.3 with a 95% CI of [49.9, 52.7] and offspring from X_SR_Y_sec_ sires: %♀ = 51.8 with a 95% CI of [50.3, 53.3]). This assay—that paired both the Paris and SR drivers with the same Y chromosomes (Y_sim_ or Y_Sec_) and in the same Y and autosomal backgrounds—demonstrates that the both the Paris and SR drivers are silenced by the same Y chromosome from D. sechellia.

#### Recombination fails to separate the Paris and SR drivers:

I next tested to see whether Paris and SR drivers can be separated by recombination. To do this, I crossed Paris males from the compound-X line to females from the SR line (X_Paris_Y_sim_ × X_SR_X_SR_, Figure S2, cross 1). This cross produced F_1_ females carrying the Paris driver on one X chromosome and the SR driver(s) on the other X chromosome (X_Paris_X_SR_). These F_1_ females were next mass mated to F_1_ males (X_SR_Y_sim_ × X_Paris_X_SR_; Figure S2, cross 2) to produce F_2_ sons (X_SR_Y_sim_ or X_Paris_Y_sim_ or X_SR&Paris_Y_sim_ or X_- -_Y_sim_, where “_ _” denotes the absence of both drivers) that were tested for sex ratio distortion by crossing one F_2_ male to 2 females (X_EVEN_X_EVEN_) from the EVEN line (400 crosses were made, Figure S2, cross 3). A total of 354 males produced offspring, and of these 30 did not produce a significant sex ratio bias. These 30 negative results could be due to binomial sampling error obscuring sex ratio distortion in smaller families (5 families were < 20 and the remaining 25 were > 65) or occur because the Paris driver is known to have incomplete penetrance (*i.e.*, some males carrying the Paris driver do not produce distorted sex ratios; Montchamp-Moreau *et al.* 2001). To check for these possibilities, brother-sister matings from the families sired by the 30 F_2_ males that did not produce significant sex ratio distortion (X_EVEN_Y_sim_ × X_EVEN_X_?_, where X_?_ denotes an X that may or may not carry a sex ratio distorter) were used (Figure S2, cross 4) to produce grand-offspring of each sire (1/2 X_EVEN_Y_sim_ and 1/2 X_?_Y_sim_). Ten male grand-offspring from these families were tested for statistically significant sex ratio distortion (*i.e.*, %♀ > 50) when crossed to dams from the EVEN line (Figure S2, cross 5). Because at least half of these male grand-offspring were not expected to carry the driver when it was present in his mother (because these dams were heterozygous: X_?_/X_EVEN_), the probability that no male grand-offspring out of 10 would carry a sex ratio distorter when it was present in the mother is ≈ 0.5^10^ < 0.001. So this protocol should effectively find cases in which a driver was present in F_2_ sires but not identified because of low statistical power or incomplete penetrance. For all of the 30 F_2_ sires that failed to produce a significantly female-biased sex ratio, I detected significant female-biased sex ratios among the offspring from the sample of his 10 male grand-offspring. These data indicate that no males were produced in the G_2_ that were not carriers of one or the other sex ratio distorter, *i.e.*, the SR or Paris SDs. This result (0 of 354 trials) can be used to construct a 95% upper bound for the map distance between the SR and Paris distorters, which is 2.0 cM. Collectively, these data suggest that the SR and Paris X-linked drivers are either allelic or very tightly linked.

## Discussion

Total egg-to-subadult mortality in the SR line was found to be insufficient to account for its observed level of its sex ratio distortion. This observation, in combination with results from the process of elimination assay [*i.e.*, step 1 (E > 50%), step 2a (biased sex ratio not transmitted mother-to-son), steps 3a, 3b, and 3c (only the paternal X predicts sex ratio of families, not the genotype of the offspring)], strongly supports the conclusion that sex ratio distortion in the SR line has a SD component. The increased sex ratio distortion in subadults compared with embryos (F > E), however, indicated that elevated male mortality or feminization also contributed substantially to the sex ratio distortion in this line. The process-of-elimination screen ruled out feminization and identified SA-ZD as a causal agent for the increased sex ratio bias in subadults compared with embryos. The operation of SA-ZD was further corroborated by the backcross X_SR_Y_EVEN_/A_SR_A_EVEN_ × X_EVEN_X_EVEN_/A_EVEN_A_EVEN_ (step 3b in Figure 3). This cross produced the full sex ratio phenotype seen in the SR line despite the fact that the X, Y, and three-fourths of the autosomes in sons were from the EVEN line, supporting the conclusion that the male mortality component of the sex ratio distortion in the SR line is due to a paternal effect.

The fact that male mortality contributed substantially to the sex ratio distortion in the SR line is proof of process that the sex-specific paternal effect required for SA-ZD to operate in *D. simulans* can be achieved. But what selective factor(s) is responsible for the evolution of this paternal-effect? At one, nonadaptive extreme, it could be an incidental, pleiotropic biproduct of selection for the SD phenotype, or also be a maladaptive artifact of evolution in a laboratory population. At the other, adaptive extreme, it could be a selectively favored phenotype (*i.e.*, SA-ZD), assuming sib-competition and sib-mating is common enough in natural populations of *D. simulans*. Because selection is expected to overcome sampling error (genetic drift) whenever the selection coefficient exceeds the reciprocal of N_e_ of the X chromosome, even very low levels of sib-competition and sib-mating would be sufficient to favor SK in *D. simulans*. Because I have not screened newly derived isolates from nature, nor have I quantified levels of potential harm from sib-competition/mating in nature, my study has only shown that the requisite sex-specific paternal-effect phenotype for SA-ZD to operate has evolved in *D. simulans* (at least in a laboratory population) and that SK can evolve as an extension of the SD phenotype. This finding moves SA-ZD from a state of theoretically possibility (Rice *et al.* 2008, 2009, 2010) to one of empirical plausibility, and it demonstrates that SA-ZD may feasibly be operating at present in natural populations of *D. simulans*.

The finding that sex ratio distortion in the SR line was not repressed by the dominant suppressor of the Winters SD driver (Nmy) nor the dominant suppressor of the Durham SD driver (Tmy) supported the conclusion that this sex ratio distorter was neither of these drivers. This conclusion is further supported by the tight linkage (≤2 cM) found here between the Paris driver (with established map location, Montchamp-Moreau *et al.* 2006) and SR driver. This linkage was too tight to include the Winters driver (Tao *et al.* 2007) nor a QTL near the white locus found to be tightly linked to the Durham driver (Dermitzakis *et al.* 2000). But the finding that both Paris and SR were fully suppressed by the same Y chromosome from *D. sechellia* indicates that SR is Paris, or a variant of it—a conclusion reinforced by their estimate complete linkage. Mercot *et al.* (1995) reported high no-hatch rates (22–25%) for a line they assayed expressing the Paris SD driver but concluded that postzygotic mortality was an unimportant contributor to sex ratio distortion because they found no correlation between brood sex ratio and brood no-hatch rate. Statistical power in this assay, however, would be expected to be low when SD produced most but not all of the sex ratio distortion. The work reported here suggests that application of the process-of-elimination assay might uncover an undetected contribution of SA-ZD to the sex ratio distortion produced by the Paris SD driver. SA-ZD may also contribute to the sex ratio distortion phenotype of other known X-linked SD elements whenever substantial egg-to-subadult mortality is associated with the driver.

The observation that the percent females in the SR liner rose from 76.5 to 83.9%♀ (or Δ_F-E_ = 7.9%) might be interpreted intuitively to imply that SA-ZD was a minor mortality factor in males; however, SD and SA-ZD operate sequentially, with SA-ZD operating only on the diminished pool of male zygotes remaining after SD has culled Y-bearing sperm. The amount of increased male mortality (relative to their sisters) required to increase the observed %F in embryos (E = 76.5%) to that seen in subadults (F = 83.9%) is 37.5%. So despite the smaller effect of SA-ZD compared with SD on the total sex ratio distortion, it nonetheless represented a substantial mortality factor in males.

SD and SA-ZD have the potential be functionally related when X-linked SD is achieved via an epigenetic modification of the Y (hereafter called a Y-linked epi-mark) that causes it to fragment or mis-segregate during meiosis and/or fail to condense during spermatogenesis. There is empirical evidence that both of these meiotic/spermatogenesis phenotypes can contribute to killing or incapacitating non-carrier sperm (*e.g.*, Montchamp-Moreau and Joly 1997; Tao *et al.* 2007). In this case, epi-marked Y chromosomes that survive spermatogenesis potentially transmit the epi-mark to embryos in the next generation and contribute to the SK phenotype of SA-ZD by producing replication, mitotic, and/or cell cycle defects, and ultimately the death of sons. This carry-over effect linking SD and SA-ZD would require the *trans*-generational transmission of the Y-linked epi-mark (*i.e.*, a form genomic imprinting). Trans-generational transmission of numerous epi-marks—that escape the normal cycle of erasure across generations—are well documented in many species (reviewed in Chong *et al.* 2007; Grossniklaus *et al.* 2013), including imprinting of the Y chromosome in flies (Maggert and Golic 2002; Menon and Meller 2010).

Suppression of SD can feasibly contribute to a transformation of SD to SA-ZD. One route to the suppression of SD is the accumulation of mutations on the Y and/or the autosomes that rescue driver-induced abnormal meiosis and/or spermatogenesis despite the presence of the Y-linked epi-mark, as opposed to suppressing the formation of the Y-linked epi-mark itself. When Y-bearing sperm are rescued via this “tolerance” route to SD suppression, functional Y-bearing sperm could transmit the Y-linked epi-mark to embryos in the next generation and feasibly cause developmental anomalies, and ultimately the death of some sons. This scenario could lead to cases in which the same locus codes for both SD and SA-ZD, with transitions between their relative contributions to sex ratio distortion accruing over evolutionary time.

Killing embryos via chromatin modification in sperm is supported by work on the cytoplasmic incompatibility (CI) phenotype produced by *Wolbachia*. Landmann *et al.* (2009) found that sperm chromatin from *Wolbachia*-infected sires of *D. simulans* mated to uninfected dams had a delayed (but not complete inhibition of) H3.3/H4 nuclear deposition during chromatin remodeling before the first mitotic division of the zygote. This delay presumably activated a cell-cycle check point in the male pronucleus that impaired the synchrony of division between male and female pronuclei, ultimately leading to embryo death. Riparbelli *et al.* (2012) reported a similar *Woplbachia*-induced CI-like phenotype in *D. bifasciata*, but in this case it killed only male offspring. Zheng *et al.* (2011) showed that down-regulating HIRA (a chaperone for H3.3 deposition) expression in the spermatozoa of *D. melanogaster* sires led to a CI-like phenotype in early-stage embryos of many of their offspring and a male-biased sex ratio (presumably because the X is more sensitive to defects in HIRA-induced chromatin remodeling compared with the Y). Collectively, these studies support the conclusion that SA-ZD could be feasibly caused by modification of the sperm chromatin that is transmitted to the zygote and induces sex-specific embryo mortality, both within and outside the context of SD.

SA-SD, with or without an association with SD, has the potential to contribute to genetic variation associated with postzygotic reproductive isolation, *i.e.*, Muller-Dobzhansky incompatibilities. X-linked SA-ZD directly selects for SK phenotype and Y-linked SA-ZD directly selects for a daughter-killing phenotype. X-linked SA-ZD may also lead to daughter-killing in hybrid crosses due to what Jaenike (2001) called “redirected drive.” In this case, an X-linked SD driver drives against itself when paired with a Y lacking a repetitive responser element (that is present at low copy number on the X). When these drivers are silenced by suppressors (on the opposite sex chromosome and/or autosomes) within a species—but this suppression breaks down in F_1_, F_2_, or backcross hybrids—then SA-ZD may contribute to hybrid inviability in a manner that exactly parallels the way that SD contributes to hybrid infertility. When the same driver causes both SD and SA-ZD, complex patterns of sex-specific hybrid inviability and infertility would be a feasible outcome.

SA-ZD also may contribute to hybrid infertility. When the cost of sib-mating is less for brothers compared to sisters, models of inclusive fitness predict that sib-mating can be favored in males and disfavored in females (Haig 1999). When sib-mating reduces female fitness, the paternal X is selected to kill or sterilize sons. This manifestation of SA-ZD could contribute to infertility in hybrid males when SA-ZD is unsuppressed in hybrid sons or their offspring, as described more fully in Rice *et al.* (2008).

Has this study provided the ultimate test for the existence of SA-ZD in *D. simulans*? No, because it is based exclusively on negative evidence from a process-of-elimination screen, rather than on positive evidence of mechanistic causation. Nonetheless, it provides compelling evidence that SA-ZD is operating in this species. The ultimate proof would require delineating the mechanism by which SA-ZD kills sons—and that the killing is favored due to sib-competition and/or sib-mating—and this has not been done here. However, a mechanism-level of investigation requires the investment of substantial resources, time, and effort, and obtaining the requisite funding requires compelling evidence that the phenomenon does, in fact, exist and needs to be explained. The process-of-elimination screen described here is meant to provide compelling—but not unequivocal—evidence for the operation of SA-ZD, which is a prerequisite to obtaining the resources needed to uncover its causation and ultimately the unequivocal proof of its existence.

The process-of-elimination screen for SA-ZD has limitations. In step 1, binomial sampling error could obscure weak SA-ZD leading to a false negative. In step 2a, a false negative could occur if endosymbionts and/or male-specific viability genes contributed predominantly to total sex ratio bias in addition to SA-ZD. A false negative could also occur in steps 2b and 3a if the EVEN line carried nuclear or cytoplasmic suppressors of SA-ZD (operating in sons)—with nontrivial levels of dominance in the case of any nuclear factors. In step 2b, a false positive might seem possible if there was latent SK in the EVEN line coded by mitochondria/endosymbionts (possibly unique to the EVEN line), but this phenotype was suppressed in sons by the Y and/or recessive genes in the EVEN line. However, this possibility is eliminated because step 3b must also be met to conclude the SA-ZD is operating. Step 3c might also seem to permit a false positive when complex interactions among the X, Y, and A from the SR line are required to produce increased male mortality. In this case, it might be argued that even though the X and Y from the SR line are present in the sons, too few matches occurred between these sex chromosomes and genes from the SR line located on the A (which are present three-fourths of the time in sons from the cross in step-3c) to produce sufficient male mortality. However, when step 3b is met (%♀ ≈ F), this unlikely possibility is eliminated. Finally, a false positive might also seem possible when multiple weak factors contribute to total sex ratio distortion (*e.g.*, a combination of weak SK, SD, and/or viability factors) because each would be too weak to meet the inequalities in steps 2a,b and 3,a,b,c—but collectively strong enough to produce measurable sex ratio distortion in the SR line. This problem is unlikely to produce a false positive, however, because equalities in steps 2b and steps 3a,c and especially step 3b must also be met to produce a false positive and these would be expected to break up any substantial summation effect (contributing to F > E) whose parts were individually too small to detect. So in sum, the process-of-elimination screen can fail to detect SA-ZD when present in the SR line when its suppressors are present in the EVEN line or when it is weak, but it should not generate false positives.

Finally, this study illustrates why SA-ZD may be far more common than presently appreciated. When observed, it is likely to be attributed to relatively uninteresting genetic variation for sex-specific viability, and not studied further, as occurred in the study by Noor and Coyne (1995). It may also be a common component of established SD drivers that is overlooked because total mortality is insufficient to account for the level of sex ratio distortion. The process-of-elimination screen described here should provide the requisite tool to uncover SA-ZD when present alone or in combination with other sex ratio distorters, so long as it is sufficiently strong.

## Supplementary Material

Supporting Information
