# Supplementary material for: An X-Linked Sex Ratio Distorter in Drosophila simulans That Kills or Incapacitates Both Noncarrier Sperm and Sons
Source: G3 (Bethesda). 2014 Jul 31;4(10):1837–48. doi: 10.1534/g3.114.013292 (PMC4199691; doi:10.1534/g3.114.013292)
Supplement: Supporting Information [file supp_4_10_1837__index.html]

An X-Linked Sex Ratio Distorter in Drosophila simulans That Kills or Incapacitates Both Noncarrier Sperm and Sons — Supporting Information 

# An X-Linked Sex Ratio Distorter in *Drosophila simulans* That Kills or Incapacitates Both Noncarrier Sperm and Sons

## Supporting Information for Rice, 2014

**Files in this Data Supplement:**

- Supporting Information - Figures S1-S2 (PDF, 509 KB)
- Figure S1 - Crosses done to examine the influence of a Y chromosome from *D. sechellia* (Ysec) on the expression of the SR and Paris sex ratio drivers. (PDF, 316 KB)
- Figure S2 - Crosses done to test for recombination between the Paris (green circles) and SR (red circles) sex ratio drivers. (PDF, 232 KB)
