# Supplementary material for: An X-Linked Sex Ratio Distorter in Drosophila simulans That Kills or Incapacitates Both Noncarrier Sperm and Sons
Source: G3 (Bethesda). 2014 Jul 31;4(10):1837–48. doi: 10.1534/g3.114.013292 (PMC4199691; doi:10.1534/g3.114.013292)
Supplement: Supporting Information [file supp_g3.114.013292_FigureS1.pdf]

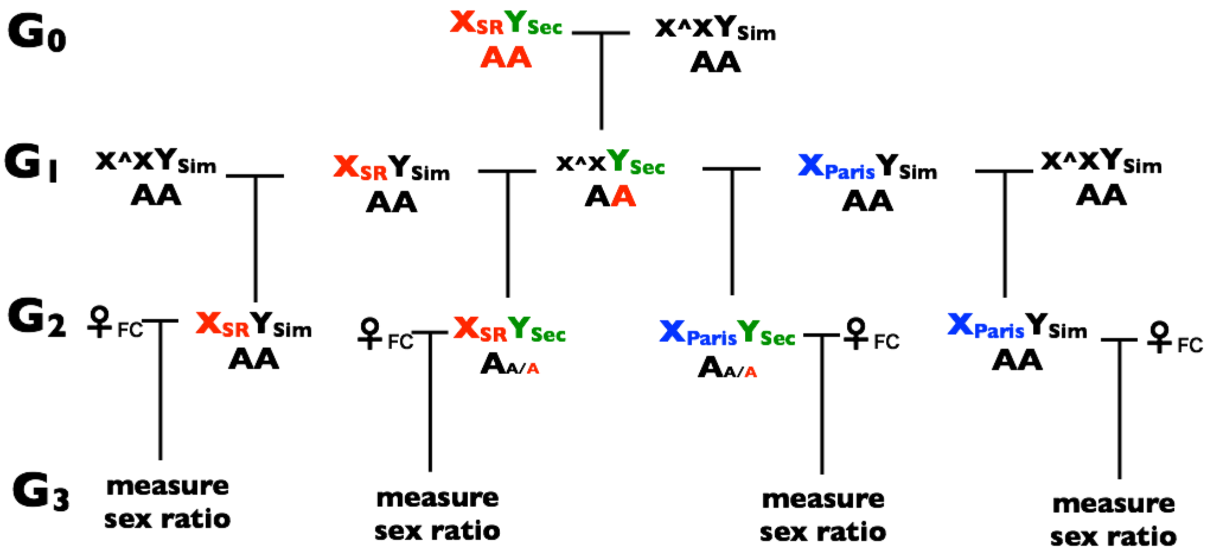

Figure S1 Crosses done to examine the influence of a Y chromosome from *D. sechellia* (Y_{sec}) on the expression of the SR and Paris sex ratio drivers. The symbol A denotes autosomes, X an X chromosome, Y_{sim} a Y chromosome from *D. simulans* and X^X a compound X chromosome. Black symbols denote chromosomes from the compound-X line and Red symbols from the SR line.
