# Supplementary material for: An X-Linked Sex Ratio Distorter in Drosophila simulans That Kills or Incapacitates Both Noncarrier Sperm and Sons
Source: G3 (Bethesda). 2014 Jul 31;4(10):1837–48. doi: 10.1534/g3.114.013292 (PMC4199691; doi:10.1534/g3.114.013292)
Supplement: Supporting Information [file supp_g3.114.013292_FigureS2.pdf]

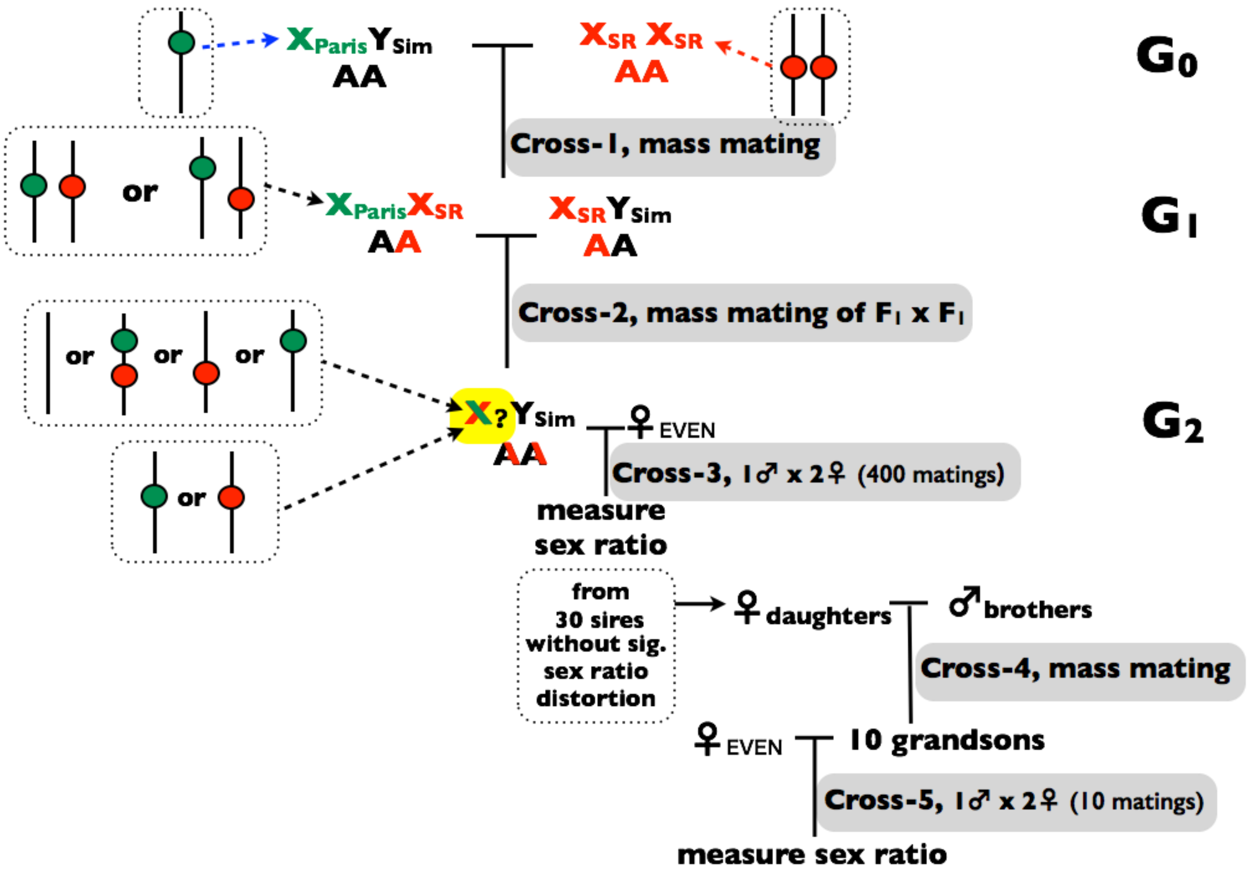

Figure S2 Crosses done to test for recombination between the Paris (green circles) and SR (red circles) sex ratio drivers.
